# Organ Preservation in Rectal Cancer: The Patients' Perspective

**DOI:** 10.3389/fonc.2019.00318

**Published:** 2019-05-10

**Authors:** Cihan Gani, Nina Gani, Sebastian Zschaeck, Fabian Eberle, Norbert Schaeffeler, Thomas Hehr, Bernhard Berger, Stefan Georg Fischer, Johannes Claßen, Stephan Zipfel, Claus Rödel, Martin Teufel, Daniel Zips

**Affiliations:** ^1^Department of Radiation Oncology, University Hospital and Medical Faculty Tübingen, Eberhard Karls University Tübingen, Tübingen, Germany; ^2^German Cancer Research Center, Heidelberg and German Cancer Consortium (DKTK), Tübingen, Germany; ^3^Gastrointestinal Cancer Center, Comprehensive Cancer Center Tübingen-Stuttgart, Tübingen, Germany; ^4^Department of Psychosomatic Medicine and Psychotherapy, University Hospital Tübingen, Tübingen, Germany; ^5^Department of Radiation Oncology, Charité Universitätsmedizin Berlin, Berlin, Germany; ^6^Department of Radiotherapy and Radiooncology, Philipps-University, Marburg, Germany; ^7^Department of Radiation Oncology, Marienhospital Stuttgart, Stuttgart, Germany; ^8^Department of Radiation Oncology, Oberschwabenklinik Ravensburg, Ravensburg, Germany; ^9^Department of Radiation Oncology, St. Vincentius-Kliniken gAG, Karlsruhe, Germany; ^10^Department of Radiotherapy and Oncology, Goethe-University Frankfurt, Frankfurt, Germany; ^11^Department of Psychosomatic Medicine and Psychotherapy, LVR-Clinic Essen, University of Duisburg-Essen, Essen, Germany

**Keywords:** psychooncology, rectal cancer, organ preservation, radiochemotherapy, shared decision making, radiotherapy

## Abstract

Organ preservation after a clinical complete response to radiochemotherapy is currently one of the most discussed topics in the management of rectal cancer. However, the patients' perspective has only been poorly studied so far. In this multicenter study, we examined 49 patients with locally advanced rectal cancer. The willingness to participate in an organ preservation study and the acceptance of the associated aspects such as intensified radiochemotherapy protocols, the need for close follow-up examinations and local regrowth rates were assessed. Attitudes were correlated with baseline quality of life parameters and psychological scales for “fear of progression”, “locus of control”, “depression”, and the “willingness to take risks”. A total of 83% of patients would consider the deferral of surgery in case of a clinical complete response (cCR). Three monthly follow-up studies and a 25% local regrowth rate are considered acceptable by 95% and 94% respectively. While 41% would be willing to exchange cure rates for a non-operative treatment strategy, a potentially more toxic radiochemotherapy in order to increase the probability of a cCR was the aspect with the lowest acceptance (55%). Psychological factors, in particular “locus of control” and “willingness to take risks”, influenced patient preferences regarding most of the assessed parameters. While in general a broad acceptance of an organ-preserving treatment can be expected, patient preferences and concerns regarding different aspects of this strategy vary widely and require specific consideration during shared decision making.

## Introduction

Preoperative radiochemotherapy (RCT) followed by radical surgery is the standard of care for locally advanced rectal cancer resulting in excellent local control rates (1). Yet long term toxicities and associated impairments of health related quality of life after trimodality treatment can be considerable and most pronounced in patients with low lying tumors when surgery requires permanent colostomy (2). At the same time, a variety of studies have shown that surgery can be omitted in carefully selected patients, when a clinical complete response is achieved after RCT (3). However, with standard RCT protocols only a minority will qualify for such a non-operative management (4). With novel, innovative RCT protocols there is a potential to at least double the fraction of patients who will achieve a complete response and therefore be candidates to omit surgery in favor of a “wait and see” strategy (5–7). The upcoming CAO/ARO/AIO-16 trial is going to test such a RCT protocol with the goal to maximize clinical complete response rates (NCT03561142). Briefly, instead of 5 weeks of RCT with concomitant 5-fluorouracil followed by surgery, patients will receive the same dose of radiotherapy; however, treatment will be intensified by the addition of Oxaliplatin to radiotherapy followed the three additional cycles of mFOLFOX6. Patients will then undergo a standardized response evaluation on day 106 after the initiation of treatment, and in case of a clinical complete response, surgery will be omitted in favor of three monthly follow-up visits with endoscopy and magnetic resonance imaging. Based on published data, approximately 25% of patients will develop local regrowths and require salvage surgery (8, 9). While a variety of studies have addressed different aspects of the non-operative approach in rectal cancer such as the optimal RCT regimen, the diagnostic accuracy of staging procedures and the oncological safety of foregoing surgery, studies reflecting the patient's perspective and preferences are sparse (10). It is well known from other entities, such as prostate cancer, where patients often have the choice between different treatment options, that the decision process is complex and influenced by factors such as cure rates, quality of life, control beliefs, fear of side effects and the desire for physical removal of the tumor by surgery rather than organ preservation by radiotherapy (11). Therefore, the present study aims to assess the patients' acceptance of different aspects of the non-operative treatment strategy in rectal cancer and correlate this with psychological and quality of life parameters. The overall goal is an improved understanding of decision making processes in rectal cancer patients who are offered a non-operative strategy.

## Materials and Methods

### Patient Recruitment

Patients with locally advanced rectal cancer who presented for preoperative RCT between January 2017 and November 2017 were included in this multicenter study. Limited German proficiency constituted a criterion of exclusion. Patients had been informed by the referring surgeon or medical oncologist about the diagnosis and the planned treatment sequence of preoperative RCT followed by surgical resection. After informed consent for preoperative RCT patients were given paper-based questionnaires covering a comprehensive set of validated instruments and additional questions specific for the planned organ-preservation trial mentioned above. The latest time-point for returning the questionnaire was the first week of RCT.

### Design and Measures

Besides demographics, patients' attitudes toward different aspects of the organ-preservation approach were assessed using Likert scales in layman's terms in German language. Likelihoods mentioned in the questions were derived from the literature (12, 13). For the purpose of this publication we translated the questions as follows:

1. Based on what you were told so far by your treating physicians, is sphincter preservation possible when surgery is performed? (Answer options: Yes - No - Possibly - no clear statement possible at this time - not yet discussed)
2. Would you participate in a clinical trial in which surgery is omitted if no tumor can been seen after radiochemotherapy? Surgery would only be performed if the tumor should regrow during follow-up. (Answer options: yes - rather yes - rather no - no - don't know)
3. A “standard” radiochemotherapy over 6 weeks results in a probability of ~10% that no tumor can be detected prior to surgery.
  - Would you accept a longer treatment with radiochemotherapy over 12 weeks if the chance that no tumor can be detected and surgery therefore can be omitted could be doubled to 20%? Assume that no increased toxicity will occur by prolonging treatment. (Answer options: yes - rather yes - rather no - no - don't know)
  - Would you accept the longer treatment if the risk for high grade toxicities such as nausea or diarrhea would increase by ~10%? (Answer options: yes - rather yes - rather no - no - don't know)
4. Now imagine, that no tumor could be detected after radiochemotherapy and surgery was omitted. In this case a close follow-up is necessary for the timely detection of a possible regrowth and making up surgery. During the first 2 years of follow-up the regime would include rectoscopies and MRI scans every 3 months. Would you participate in an organ-preservation trial despite this follow-up regime? (Answer options: yes - rather yes - rather no - no - don't know)
5. As said, there is the possibility that a tumor regrowth is detected during follow-up and that surgery has to be performed. Would you accept such a likelihood of 25% that surgery has to be performed? (Answer options: yes - rather yes - rather no - no - don't know)
6. The chances for cure in patients with tumors that respond very well and cannot be detected after radiochemotherapy are ~90% if surgery is performed. How high does the cure rate have to be at least, so that you would accept the omission of surgery, if no tumor can be detected after radiochemotherapy. (Answer options: At least 90% and therefore at least as high as with surgery - slightly lower, e.g., 88%–Clearly lower, e.g., 80%–Don't know / Don't understand the question.)

The EORTC Quality of life C30 (EORTC-QLQ-C30) questionnaire is a 30 item questionnaire to determine various dimensions of health related quality of life, designed for the use in cancer patients (14). The EORTC-QLQ-C30 covers five functional measures (physical, role, emotional, social, cognitive) and eight symptoms (fatigue, pain, nausea/vomiting, appetite loss, constipation, diarrhea, insomnia, dyspnea). Furthermore, global health/QOL and financial problems are assessed. The current study cohort's quality of life was compared with the EORTC reference cohort for colorectal cancer (male age group 60–69 years) and published values for the German general population (15).

Fear of progression was assessed using the 43 item “FoP-Q” (16). The 43 items result in the five dimensions “affective reactions”, “partnership/family”, “occupation”, “loss of autonomy”, and “coping with anxiety”. The score for each dimension can range from 1 to 5. Internal consistency of the FoP-Q is high (Cronbach's *a* = 0.95), as is the retest reliability (rtt = 0.94) (16).

The KKG-questionnaire measures control beliefs, whether individuals assign life circumstances to internal or external (e.g., physicians) circumstances. A third dimension is the “fatalistic” locus of control, indicating an individual's belief that fate and luck have a crucial impact on their health. The entire questionnaire included 21 items, seven per dimension with score ranging from 7 to 42 per scale and higher score indicating higher control beliefs. Retest reliability for the individual dimensions ranges between 0.74 and 0.78, internal consistency between 0.64 and 0.77. (17). The “Patient Health Questionnaire 8” (PHQ-8) was used to measure depressive symptoms with 8 items and a maximum summed score of 24 points. A score of 10 or higher is considered as major depression, a score of 20 or higher as severe depression. In the absence of appropriate tools to assess the willingness to take risks we developed a Likert scale based question in German language. The single item “How high is your willingness to take risks” ranges from “1– not at all willing to take risks” to “7–Very willing to take risks.” A patient with a score of “5” or higher was defined as having a high willingness to take risks.

### Statistical Analysis

Patient characteristics are presented by the mean values with standard deviations (SD) or frequencies. Unpaired independent variables were compared by using an independent samples *t*-test or Mann-Whitney's U-test. Chi-square testing was performed to compare frequencies. An ordinal regression was performed to assess relationships between Likert scale based items mentioned above and continuous variables. “Acceptance” was defined as given if either “yes” or “rather yes” was chosen as an answer in the Likert scale based items. Statistical analysis were performed using IBM SPSS, Version 24 (IBM Corporation, Armonk, NY, USA) and GraphPad Prism, Version 6, Graphpad Software, La Jolla, California, USA).

## Results

### Sample Characteristics

A total of 49 rectal cancer patients (*n* = 13 female, *n* = 36 male) who presented for multimodality treatment consisting of preoperative radiochemotherapy and subsequent surgical resection were included in this study. Patients were referred to the radiation oncologist after the diagnosis and an outline of the multimodal treatment had been communicated by the referring surgeon or oncologist. Table 1 shows an overview of patient related parameters. A total of 41% of the patients had previously been told that sphincter preservation would be possible when surgery is performed. In 45% of the patients, sphincter preserving surgery was considered only “possibly” feasible and “not possible” in 2%. Twelve percent reported that odds for sphincter preservation were not discussed at all so far.

**Table 1:** Patient characteristics, demographic and psychological data.

	***n***	**%**
	**49**	**100**
Age median (range)	61 (37–79)	
Gender
Female	13	27
Male	36	73
Martial Status
Married/in partnership	37	76
Windowed/not in partnership	12	24
Highest Degree Of Education
Elementary school/secondary modern school	20	41
Middle school	17	35
High school	7	14
Postgraduate/University degree	4	8
Missing	1	2
Employment
Employed	26	53
Retired	22	45
missing	1	2
Residence (Inhabitants)
Village (<5,000)	24	49
Small town (<20,000)	7	14
Medium sized town (<100,000)	5	10
Major city (>100,000)	13	27
	**Mean**	**SD**
Fear Of Progression
Affective reactions	2.49	0.73
Partnership/family	2.31	0.65
Occupation	1.97	0.97
Loss of autonomy	2.34	0.75
Coping with anxiety	3.46	0.58
Locus of Control
Internality	25	5.3
Social externality	25	5.4
Fatalistic externality	23	6.3
Willingness to take risks	4	1.5

*Scales range from 1 to 5 for the fear of progression scale, 7–42 for locus of control and 1 to 7 for the willingness to take risks*.

### Quality of Life and Psychological Scores

**Figure 1:**
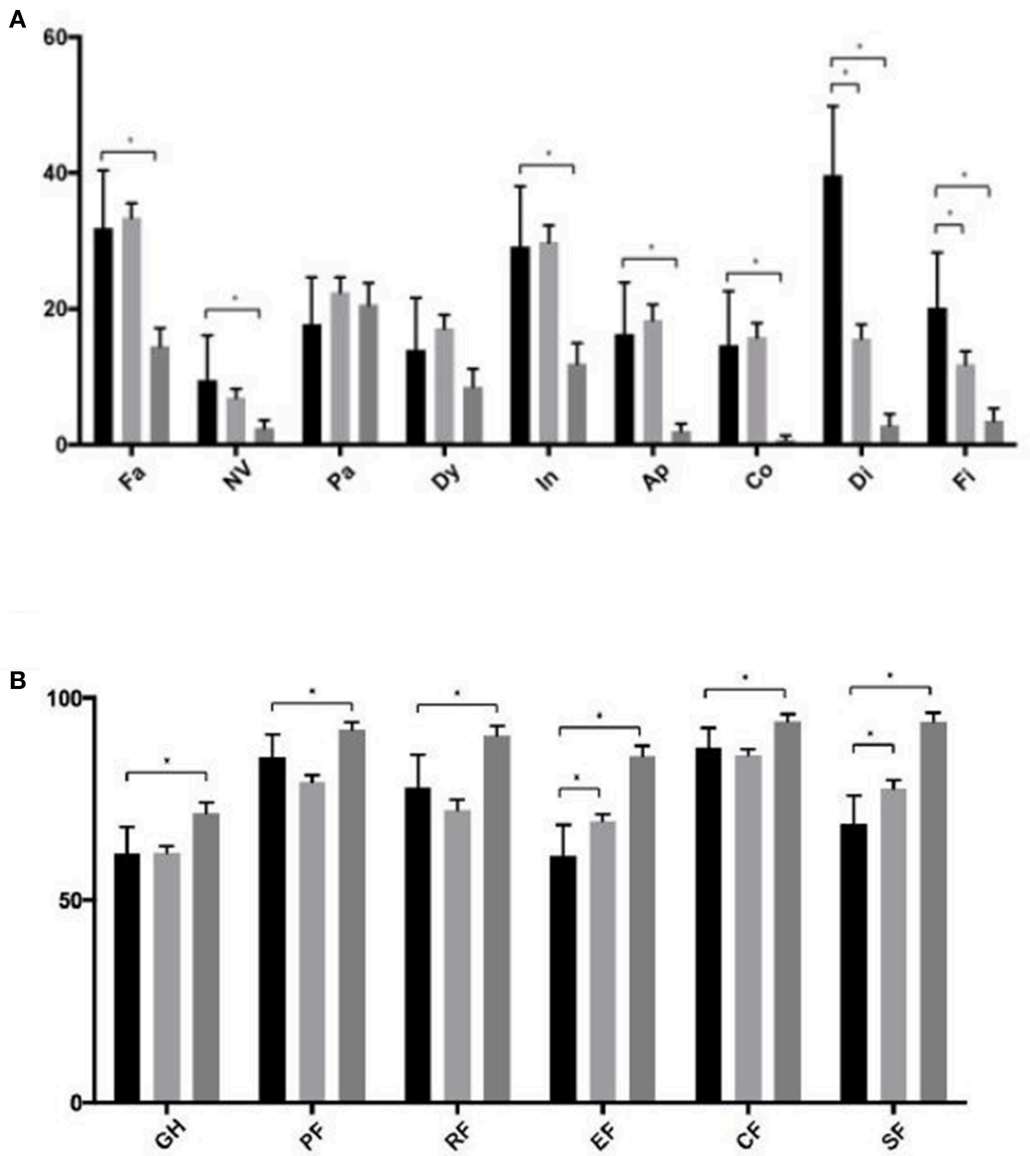
**(A,B)** Symptom and function sub-scales of the EORTC QLQ-C30 subscales. Black bars represent patients from the current study, light gray the EORTC colorectal cancer reference group and dark gray the German general population. Asterisk indicate a *p* < 0.05. Fa, Fatigue; NV, Nausea/Vomiting; Pa, Pain; Dy, Dyspnea; In, Insomnia; Ap, Appetite loss; Co, Constipation; Di, Diarrhea; Fi, Financial problems; GH, Global health status; PF, Physical Function; RF, Role Function; EF, Emotional Function; CF, Cognitive Function; SF, Social Function.

In our cohort, overall quality of life according to EORTC QLQ-C30 was comparable with the EORTC colorectal cancer reference group but significantly lower compared with the German general population (61.5 vs. 71.6, *p* = 0.001). Compared with the latter, patients in our cohort had significantly worse function and symptom scores on all scales except for “Dyspnea” and “Pain”. The EORTC cohort had significantly higher function scores for “Emotional function” and “Social function”, while “Diarrhea” and “Financial concerns” were expressed more often in our cohort (Figure 1).

The median depression score in our cohort according to PHQ-8 was 4 (range 0–15) with 15% of patients meeting criteria for major depression (18). Patients who were told that sphincter preservation will be possible in case of surgery had significantly lower mean scores on the PHQ-8 scale compared with patients whose sphincter preservation with surgery was unsure or considered impossible (3.56 vs. 5.74, *p* = 0.048).

### Acceptance of Individual Aspects of the Non-operative Approach

**Figure 2:**
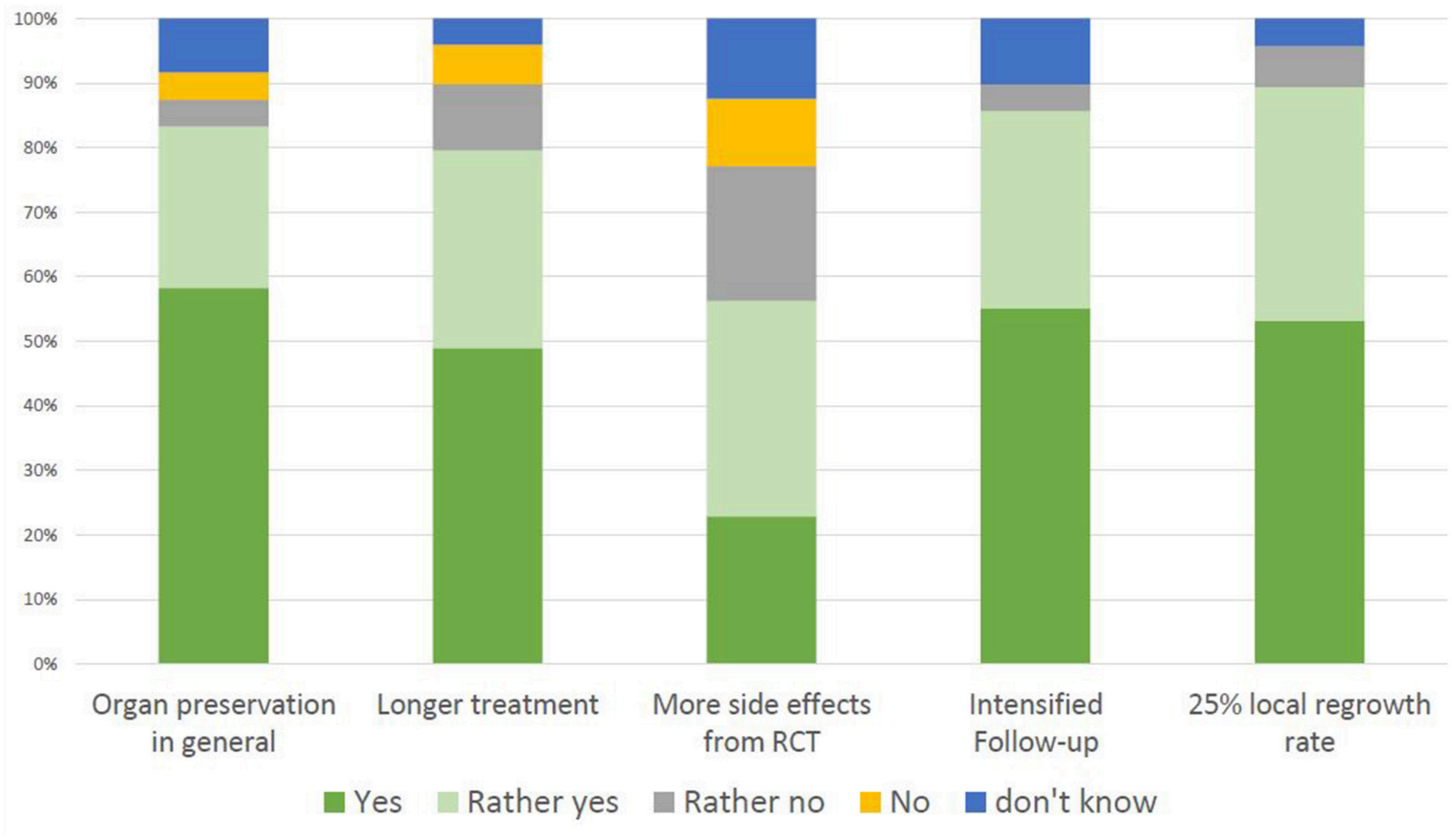
Patient acceptance of individual aspects of an organ-preservation trial. “Acceptance” is defined as the summed percentage of the answers “yes” and “rather yes”.

A total of 83% of the patients would accept the deferral of surgery in case of a clinical complete response after RCT in favor of salvage surgery should a local regrowth develop during surveillance, Figure 2. Patients who would not accept this strategy had a significantly higher PHQ-8 score for depression (10.67 vs. 4.41, *p* = 0.021) and also in the “affective reactions” subscale in the PA-F questionnaire (3.01 vs. 2.44, *p* < 0.001). The likelihood of sphincter preservation during surgery had no impact on the general willingness to participate in an organ-preservation trial (*p* = 0.547). A longer but equally toxic RCT regimen would be accepted by 80%, on ordinal regression low scores for Internality (OR 1.2, 95% CI 1.03–1.39, *p* = 0.016) and high scores for Social Externality (OR 1.21, 95% CI 1.05–1.41, *p* = 0.010) and a high willingness to take risks (OR 17.56, 95% CI 2.85–108.21, *p* = 0.002) predicted the acceptance of this aspect. Similarly, a higher score for Social Externality (OR 1.18, 95% CI 1.03–1.35, *p* = 0.020) and a high willingness to take risks predicted the acceptance of a more toxic RCT protocol (OR 32.35; 95% CI 5.24–199.79, *p* < 0.001). Only 4% would refuse participation in an organ preservation protocol due to three-monthly MRIs and rectoscopies. For 6.4% of patients a local regrowth rate of 25% is considered unacceptable. For these two questions no ordinal regression was performed due to the low rate of negative replies. Equal cure rates as with immediate radical surgery is a prerequisite for non-operative management for 55% of patients. However, 30% would accept a long-term cure rate of 88% instead of 90 and 11% would still prefer a non-operative approach if this was associated with a cure rate of 80% instead of 90%. A higher fear of progression was seen in patients who would accept lower cure rates (OR 1.41, 95% CI 1.01–1.97, *p* = 0.42). The latter observation was mostly driven by the subscale for “affective reactions” with a mean score of 2.7 for patients who would accept lower cure rates compared with a score of 2.3 for those who would not (*p* = 0.042) as well as the subscale “partnership/family” (mean scores 2.6 vs. 2.1, *p* = 0.005).

## Discussion

Our study is one of the first to explore patients' attitudes and preferences on various aspects of a non-operative management strategy in rectal cancer. The overall goal was to gain a deeper understanding of factors that influence patients' decision making process for or against such a treatment. Our cohort was representative in terms of baseline symptoms, functioning and global health status compared with the corresponding EORTC reference group (19). The only symptom with a higher score in our cohort was “diarrhea”, which might be due to the inclusion of early and late stages of disease of both colon and rectal cancer in the EORTC reference cohort. We observe a large acceptance for the omission of surgery in case of a clinical complete response, which is an expected finding. Yet still 16% of the patients in our cohort could not imagine to participate or were undecided. Preceding studies on patient preference in breast cancer and prostate cancer have consistently shown that a considerable number of patients believe that radical surgery is the only treatment that will cure their disease (20, 21). It is therefore possible that such thoughts play a crucial role in a patient's decision making process when being confronted with an organ preserving treatment strategy in rectal cancer. Our study identified two factors that were significantly associated with the acceptance of both a longer and a slightly more toxic RCT treatment: A higher willingness to take risks and a higher external locus of control. The slightly more toxic radiotherapy protocol was the factor with the highest potential to prevent patients from participation in an organ-preservation trial. This observation is likely associated with long known fears and misperceptions of radiotherapy (22). Already in the 1970s a study by Peck et al. showed that the majority of patients in reference to radiotherapy “believed that requiring radiotherapy was bad news” and that “radiotherapy is inherently damaging” (23). Similarly, a survey in Canada published four decades later still revealed that side effects caused by radiotherapy both during and after treatment were considered a major concern in ~80% of patients (24). This underlines the importance of a detailed discussion of the likelihood and severity of radiotherapy related side effects between the patient and physician. In this context it should be noted that the RCT protocol in the upcoming CAO/ARO/AIO-16 trial is adopted from the preceding CAO/ARO/AIO-04 trial. In that trial treatment compliance and toxicity was excellent despite an intensified chemotherapy protocol with 94% of patients receiving the prescribed dose of radiotherapy and 85% the full dose of chemotherapy (25). We therefore do not expect that the planned RCT regime of the CAO/ARO/AIO-16 trial will hamper study recruitment due to toxicity concerns.

When we designed the questionnaire we had hypothesized that an expected local regrowth rate of 25% and follow-up regime with three-monthly imaging and endoscopic studies would be considered unacceptable by a considerable fraction of participants. Furthermore, we had expected to see a correlation of “fear of progression” with the acceptance of these parameters. Fear of progression or recurrence has been described as a major issue in cancer survivors with unmet needs in a systematic review (26). However, only 4% (follow-up exams) and 6% (local regrowth rate) of the participants in the present study consider these aspects an obstacle for the participation in an organ-preservation protocol. A possible explanation for this is that the chance of omitting surgery weighted more than concerns associated with follow-up studies and local regrowth rates. At a closer look at the single items of the fear of progression scale, the item reflecting the greatest fear on the “affective reactions” scale was “I am afraid of severe medical treatments in the course of my illness.” supporting this explanation. Interestingly, the general willingness to participate in an organ-preservation trial was independent of the chances for sphincter preservation during surgery. This further supports the hypothesis that fear of surgery also plays a crucial role in the patients' decision making process (27).

Concerns regarding the safety of an organ-preservation approach include the possibility of synchronous distant spread with local regrowth resulting in an incurable disease. Based on published data available, the non-operative approach appears oncologically safe without compromising long-term cure rates in patients who develop local regrowth's and undergo salvage surgery (28). In a recent series of 100 patients managed non-operatively only two patients developed local regrowth's with distant failures. Assuming a worst case scenario in which both distant failures originated from the local regrowth and could have been prevented by immediate surgery after RCT, the oncological risk is ~2–3% (12). We used this rate and a hypothetical 10% decrease in cure rate to estimate the patients' willingness to exchange long-term cure rates for the chance of organ-preservation. More than 40% of the participants were willing to accept lower long-term cure rates for the omission of surgery in case of a clinical complete response. Data on time-trade-off preferences in rectal cancer patients is sparse. Schmidt et al. included both lung and colorectal cancer patients of all stages in a study investigating therapy preferences from the patient perspective and found that patients with colorectal cancer put more weight on health related quality of life than on life expectancy than lung cancer patients. A low baseline health related quality of life was a predictor for the willingness to trade-off survival for organ preservation which is in line with reports from studies with oncological and non-oncological patients (29, 30).

Patients willing to accept lower long-term cure rates had a significantly higher score in the fear of progression scale, a finding which appears counterintuitive on first sight. However, several items of the PA-F scale address concerns of functional impairments caused by the disease itself or the treatment. Patients who were willing to trade-off survival for organ preservation expressed more concerns in items related to family, sexuality and profession and therefore a decrease HRQoL after radical treatment providing a plausible explanation for higher “fear of progression score” in these patients.

The current study constituted a “mind-game” which can be considered a limitation of the trial since we cannot rule out that the patients would have chosen different answers if they had truly been in a setting to decide for or against an intensified RCT regime with the goal for non-operative management. Furthermore, the small sample size and potential cultural differences need to be considered when extrapolating the results of our study (31).

In summary, our study shows a high acceptance of the organ-preservation approach among rectal cancer patients. Yet at the same time we demonstrate a broad variety in patient preferences regarding individual aspects of this treatment strategy. Particularly patients with a high level of external locus of control and high willingness to take risks will also accept longer and potentially more toxic RCT protocols. Furthermore, fear of radiotherapy related toxicity might hamper recruitment in studies investigating intensified treatment protocols. Most importantly our study underlines the importance of a thorough and realistic explanation of possible treatment related side effects and the exploration of a patient's prioritization regarding quality of life, functioning and oncological safety.

## Ethics Statement

As stated in the manuscript, the need for an IRB approval was waived by the institutional review board of the Medical Faculty in Tübingen/Germany, since only anonymized data was collected und studied in this non-interventional study.

## Author Contributions

CG, NG, DZ, MT, NS, CR, and StZ: Conceptualization. CG, SeZ, FE, TH, BB, SF, JC, and CR: Data collection and patient recruitement. CG, NG, StZ, CR, MT, NS, and DZ: Data analysis and interpretation. CG, NG, StZ, MT, and DZ: Writing.

### Conflict of Interest Statement

The authors declare that the research was conducted in the absence of any commercial or financial relationships that could be construed as a potential conflict of interest.
